# Male-Specific Effects of β-Carotene Supplementation on Lipid Metabolism in the Liver and Gonadal Adipose Tissue of Healthy Mice

**DOI:** 10.3390/molecules30040909

**Published:** 2025-02-15

**Authors:** Yeonsoo Oh, Jinsol Kim, Yoon Jung Park, Yuri Kim

**Affiliations:** 1 Department of Nutritional Science and Food Management, Ewha Womans University, Seoul 03760, Republic of Korea; helloimoys@naver.com (Y.O.); wlsthf4519@naver.com (J.K.); park.yoonjung@ewha.ac.kr (Y.J.P.); 2 Graduate Program in System Health Science and Engineering, Ewha Womans University, Seoul 03760, Republic of Korea

**Keywords:** β-carotene, sex differences, β-carotene cleavage enzyme, estrogen receptor, lipid metabolism, liver, adipose tissue

## Abstract

Biological sex is a fundamental determinant of physiological differences, including metabolic processes and disease susceptibility. β-carotene (BC), a provitamin A carotenoid, is known for its health benefits, but its sex-specific effects on its metabolism remain largely unexplored. This study investigated male and female BALB/c mice receiving BC or vehicle control via oral gavage for 11 weeks. Hepatic and circulating lipid levels, serum retinol, and the expression of BC cleavage enzymes (*Bco1* and *Bco2*) and estrogen receptors (*Esr1* and *Esr2)* in the liver and gonadal fat were analyzed. BC supplementation increased the hepatic *Bco1* and *Bco2* expression in males, accompanied by higher serum retinol, while downregulating expressions of these enzymes in male gonadal fat. Additionally, BC supplementation significantly reduced gonadal fat mass and adipogenic gene expression in males, with *Cebpa* and *Esr1*/*Esr2* positively correlated, suggesting a role for estrogen receptor signaling in adipogenesis. These findings demonstrate that BC exerts sex- and tissue-specific effects on lipid metabolism, with strong regulatory interactions between BC metabolism, lipid homeostasis, and sex hormone signaling in males. The results provide novel insights into the mechanisms underlying sex-dependent differences in lipid metabolism following BC supplementation, with potential implications for metabolic health and disease prevention.

## 1. Introduction

Biological sex is a fundamental determinant of physiological differences, including metabolic processes and disease susceptibility, in various organisms. Extensive research has revealed the profound influence of sex on diverse physiological states, including lipid metabolism, metabolic diseases, and cancer [1,2,3,4,5]. Notably, the risk of cardiovascular disease and obesity-related complications in premenopausal women is lower than that in men possibly because of the sex-specific modulation of lipid metabolism [4,5]. Furthermore, the risk for various cancer types in females is lower and their prognosis is better [3]. These observations highlighted the necessity of considering sex as a crucial biological variable in biomedical research because it can profoundly affect an individual’s risk for developing certain conditions and their response to therapeutic interventions.

Although sex hormones, such as estrogen and testosterone, have been traditionally associated with reproductive functions, they elicit diverse physiological effects by modulating lipid and glucose metabolism [4,6,7]. For example, the liver exhibits sexual dimorphism in its metabolic capacity, and hepatic receptors for sex hormones such as estrogen participate in regulating lipid and glucose homeostasis [7,8]. Specifically, estrogen signaling regulates hepatic lipid metabolism by reducing triglyceride (TG) production and enhancing fatty acid oxidation upon activation [4].

The distribution of adipose tissue is influenced by sex; females typically have a higher proportion of subcutaneous fat than males [5]. In the adipose tissue, the functions of estrogen receptors (ERs) and androgen receptors (ARs) vary between subcutaneous and visceral depots, thereby affecting adipogenesis, lipid metabolism, and adipokine secretion [7,9]. This sex-specific pattern of fat distribution has considerable implications for sex-specific differences in metabolic health because visceral adiposity is strongly associated with an increased risk of metabolic disorders such as insulin resistance and cardiovascular disease [4]. Therefore, biological sex should be considered a potential factor in metabolic responses, because differences in sex hormone signaling can lead to crucial sex dimorphisms in metabolic regulation and disease susceptibility.

Nutritional factors may contribute to sex-specific metabolic profiles [10]. For instance, β-carotene (BC), a provitamin A carotenoid, has been widely explored because of its various health benefits, including antioxidant [11], anti-obesogenic [12], and immunomodulatory effects [13]. However, potential sex-specific differences in BC metabolism and their influence on lipid metabolism remain largely unexplored. Several studies have suggested that males and females may vary in BC bioavailability and conversion efficiency to vitamin A; furthermore, the metabolic effects of BC are potentially influenced by hormonal, genetic, and physiological factors [14,15]. Given the intricate interplay between sex hormones and nutrient metabolism, studies should investigate the sex-specific effects of BC supplementation on lipid metabolism in key metabolic tissues, such as the liver and adipose tissues.

This study aimed to explore the sex-specific effects of BC supplementation on lipid metabolism in mouse liver and gonadal white adipose tissue. By elucidating the potential sex-dependent mechanisms underlying the metabolic effects of BC, this research helped further explain the relationship between nutrition, sex hormones, and metabolic regulation. The findings may contribute to developing personalized nutritional and therapeutic interventions depending on individual sex-specific needs. Thus, precision medicine can be improved, and better health outcomes for both sexes can be promoted.

## 2. Results

### 2.1. Male-Specific Effects of BC on Hepatic Lipid Accumulation

Publicly available datasets were obtained and DEGs were identified in liver tissues (GSE98846) from the GEO database to elucidate the sex differences of the impact of BC on hepatic gene expression. In the liver, 487 DEGs were detected in mice fed with a control diet and 469 DEGs in BC-supplemented mice when comparing the gene sets between sexes (Supplementary Figure S1A). GO analysis was conducted using 86 DEGs specific to the BC-supplemented condition in the comparison of males and females (Supplementary Figure S1B). The GO terms enriched for the up-regulated DEGs in the liver were associated with steroid/lipid metabolism processes (Supplementary Figure S1(Ba)). This finding suggested the potential molecular effectiveness of BC in regulating hepatic lipid metabolism. Based on this preliminary investigation, we hypothesized that BC might elicit sex-specific responses in hepatic lipid metabolism pathways.

**Figure 1:**
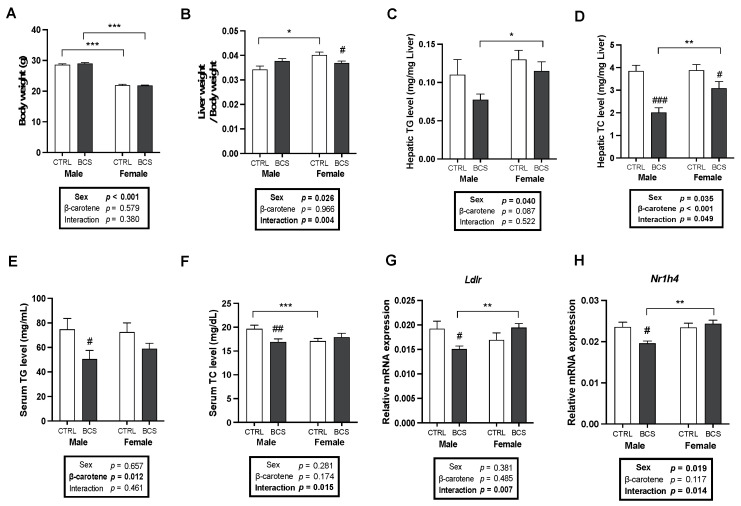
Sex differences in body and liver weight, lipid profiles, and cholesterol-metabolism-related hepatic gene expression upon BC supplementation. (**A**) Body weights and (**B**) liver weights/body weights after 11 weeks of intervention. Hepatic TG (**C**) and TC (**D**) levels were measured (n = 12 /group). Serum levels of (**E**) TG and (**F**) TC were analyzed (n = 12/group). mRNA expression of (**G**) *Ldlr* and (**H**) *Nr1h4* (encoding FXR) in the liver was analyzed by RT-qPCR analysis (n = 12/group). *Gapdh* was used as a loading control. All data were analyzed using two-way ANOVA with Tukey’s multiple comparisons. Results are presented as mean ± SEM. * for between sexes and # for between treatment groups (*, # *p* < 0.05, **, ## *p* < 0.01, ***, ### *p* < 0.001 in Student’s *t*-test). BCS, group supplemented with 15 mg/kg body weight of β-carotene; CTRL, control group; TC, total cholesterol; TG, triglycerides.

BC supplementation did not significantly influence body weight across sexes (Figure 1A). However, a significant interaction between sex and BC treatment was observed in terms of liver weight, suggesting potential sex-specific hepatic responses to BC (Figure 1B). BC supplementation decreased the hepatic TG levels in males and significantly reduced the hepatic total cholesterol (TC) levels in both sexes (Figure 1C,D). Interestingly, TC levels of male mice were lower than those of female mice with a significant interaction between sex and BC treatment (Figure 1D). Serum TG and TC levels were assessed to investigate serum lipid alterations by BC supplementation. The serum TG levels of the male BCS group were significantly lower than those of the male CTRL group (Figure 1E). Additionally, serum TC levels significantly decreased in the male BCS group compared with that in the male CTRL group; a significant interaction was also observed between sex and BC supplementation, indicating male-specific reduction (Figure 1F). These findings suggested that BC might exert sex-specific effects on BC metabolism. BC supplementation persistently reduced hepatic and serum cholesterol levels in males and as such the expression levels of genes related to cholesterol metabolism were explored. BC supplementation significantly down-regulated the mRNA expression of low-density lipoprotein receptor (*Ldlr*) and nuclear receptor subfamily 1 group H member 4 (*Nr1h4*), which encodes the farnesoid X receptor (FXR), in males; this expression was lower in the male BCS group than in the female BCS group (Figure 1G,H). A significant interaction was found between sex and BC supplementation for mRNA expression levels of *Ldlr* and *Nr1h4*, indicating sex-dependent BC effects on cholesterol metabolism in males (Figure 1G,H).

### 2.2. Male-Specific Increases in Hepatic BC Metabolism and Mobilization upon BC Supplementation

**Figure 2:**
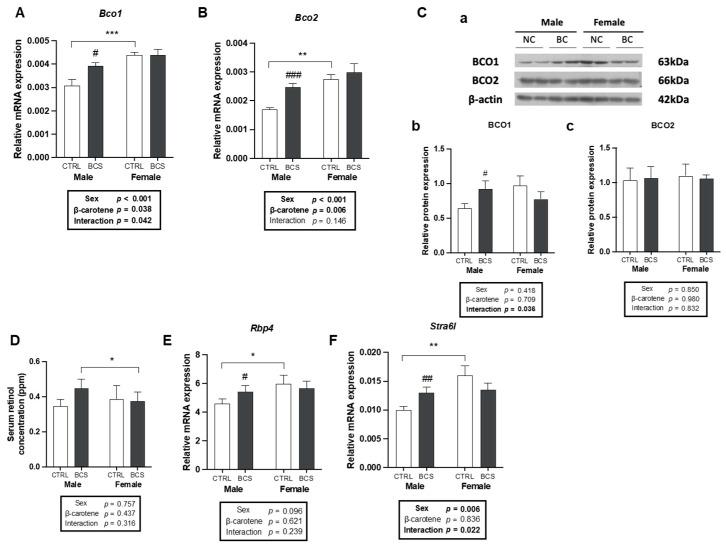
Male-specific increases in hepatic BC metabolism and mobilization upon BC supplementation. mRNA expression of (**A**) *Bco1* and (**B**) *Bco2* in the liver was analyzed by RT-qPCR analysis (n = 12/group). (**C**) Protein expression of BCO1 and BCO2 was analyzed by Western blot. Representative bands (**a**) and quantifying protein expression of (**b**) BCO1 and (**c**) BCO2 are presented (n = 12/group). All blots were normalized to β-actin. (**D**) HPLC analysis of serum retinol levels in male and female mice (n = 8/group). mRNA expression of (**E**) *Rbp4* and (**F**) *Stra61* in the liver is presented (n = 12/group). *Gapdh* was used as a loading control for all RT-qPCR analyses. All data were analyzed using two-way ANOVA with Tukey’s multiple comparisons. Results are presented as mean ± SEM. * for between sexes and # for between treatment groups (*, # *p* < 0.05, **, ## *p* < 0.01, ***, ### *p* < 0.001 in Student’s *t*-test). BCO1, β-carotene-15,15′-oxygenase; BCO2, β-carotene-9,10′-oxygenase; BCS, group supplemented with 15 mg/kg body weight of β-carotene; CTRL, control group; HPLC, high-performance liquid chromatography.

To better understand hepatic BC metabolism, the expression of BC cleavage enzymes was analyzed. BCO1 and BCO2 are critical enzymes that cleave BC to various metabolites, including retinoic acid [16,17]. BC administration significantly increased the *Bco1* expression exclusively in males, exhibiting a significant interaction between sex and BC supplementation (Figure 2A). Similarly, BC supplementation up-regulated the *Bco2* expression only in males, with a significant sex difference only in the BCS groups without the interaction between sex and BC supplementation (Figure 2B). Consistent with the male-specific enhancement of *Bco1*, BC up-regulated BCO1 protein levels, with a significant interaction between sex and BC; this finding indicated a potential male-dependent regulation of BCO1 expression (Figure 2(Cb)). Conversely, BCO2 protein levels remained unchanged upon BC supplementation in both sexes (Figure 2(Cc)).

Retinol, a biologically active vitamin A form, is generated from retinyl aldehyde through the conversion from BC by BCO1 [16]. To substantiate the increased activation of BCO1 observed in males following BC supplementation, circulating serum retinol levels were quantified via HPLC. Although no significant differences were detected within or between the male and female groups, the retinol concentration of the male BCS group was higher than that of the CTRL group (Figure 2D). The mRNA expression of a retinol carrier protein *Rbp4* and the expression of its receptor *Stra6l* were examined to assess BC mobilization. BC supplementation increased the mRNA expression levels of *Rbp4* and *Stra6l* only in males (Figure 2E,F). A significant interaction between sex and BC was detected in *Stra6l*, but not in *Rbp4*. Therefore, males might have enhanced BC mobilization in the liver, but no significant differences in circulating retinol were found upon BC supplementation.

### 2.3. Male-Specific Up-Regulated Expression of ERs by BC in the Liver upon BC Supplementation

**Figure 3:**
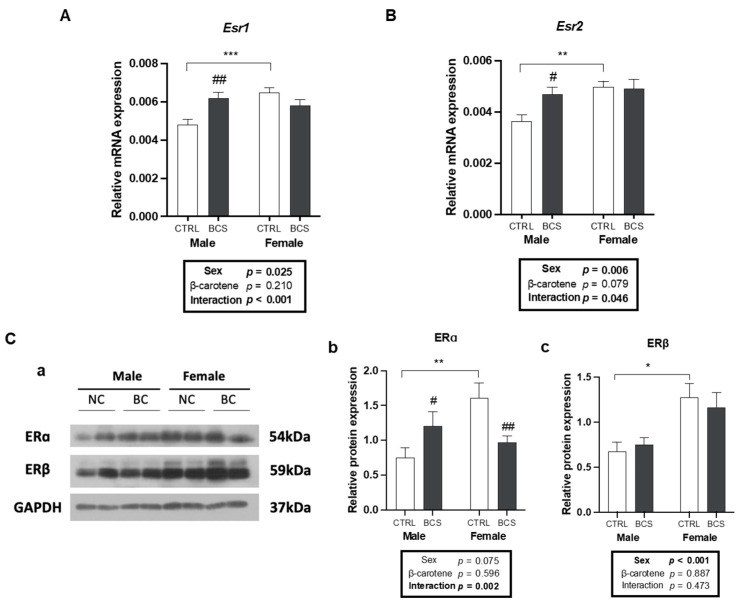
Male-specific up-regulated expressions of ERs by BC in the liver upon BC supplementation. mRNA expression of (**A**) *Esr1* (encodes ERα) and (**B**) *Esr2* (encodes ERβ) in the liver was analyzed by RT-qPCR analysis (n = 12/group). *Gapdh* was used as a loading control. (**C**) Protein expression of ERɑ and ERβ was analyzed by Western blot. Representative bands (**a**) and quantifying protein expression of (**b**) ERɑ and (**c**) ERβ are presented (n = 12/group). All blots were normalized to GAPDH. All data were analyzed using two-way ANOVA with Tukey’s multiple comparisons. Results are presented as mean ± SEM. * for between sexes and # for between treatment groups (*, # *p* < 0.05, **, ## *p* < 0.01, *** *p* < 0.001 in Student’s *t*-test). BCS, group supplemented with 15 mg/kg body weight of β-carotene; CTRL, control group; ER, estrogen receptor.

The potential sex-specific effects of BC on mRNA and protein ER expression were investigated. As expected, basal *Esr1* and *Esr2* expression levels were higher in females than in males. However, BC supplementation significantly increased the mRNA expression levels of *Esr1* and *Esr2* only in males, exhibiting a significant interaction between sex and BC supplementation (Figure 3A,B). At the protein level, BC supplementation up-regulated the estrogen receptor (ERα) in males and down-regulated the ERα expression in female subjects; furthermore, a significant interaction was observed between sex and BC supplementation (Figure 3(Cb)). Conversely, ERβ expression levels differed only between the CTRL groups across sexes (Figure 3(Cc)). Therefore, these results indicated the sex-dependent effects of BC in modulating the ER expression, with a more potent regulatory effect on males.

### 2.4. Male-Specific Potential Correlations of Nr1h4, Bco1, and Esr1 Expression

**Figure 4:**
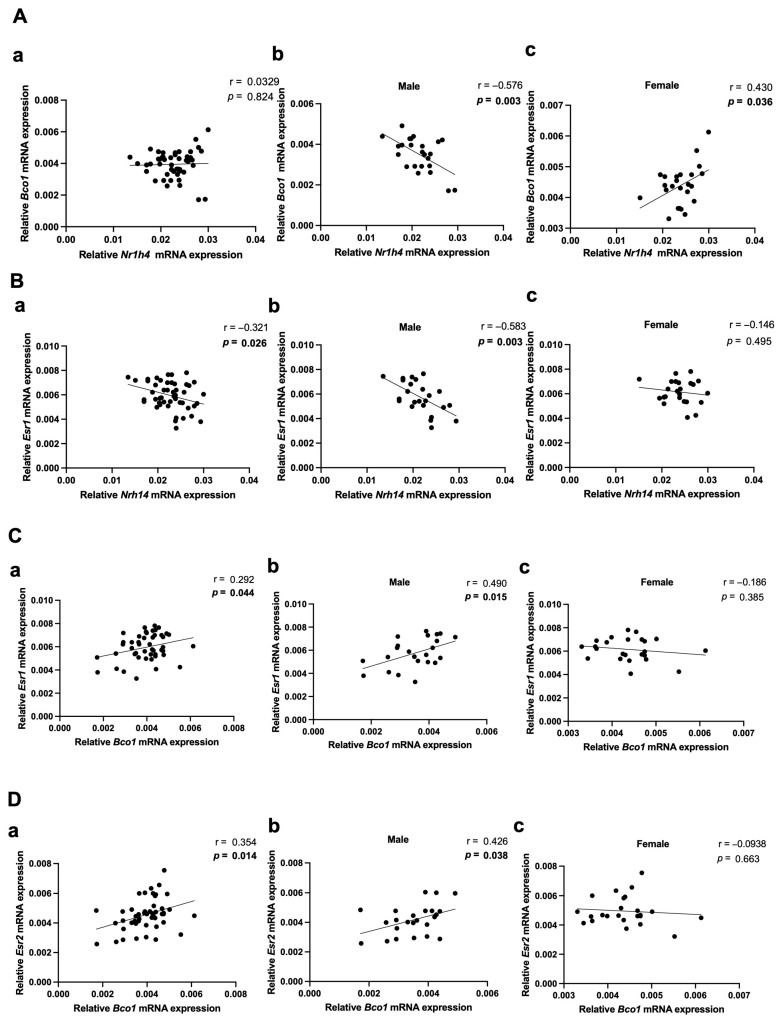
Male-dependent potential correlations of *Nr1h4*, *Bco1*, and *Esr1* expressions. Correlation of the mRNA expressions of *Nr1h4* (encoding FXR) with (**A**) *Bco1* and (**B**) *Esr1* mRNA expression (n = 12/group). Correlation of *Bco1* mRNA expressions with (**C**) *Esr1* (encodes ERɑ) and (**D**) *Esr2* (encodes ERβ) (n = 12/group). Pearson’s correlation coefficient was employed for statistical analysis. Correlations were observed for the entire dataset as well as separately for male and female mice; (**a**) male + female, (**b**) male, and (**c**) female mice. Statistical significance was defined as *p* < 0.05. The 95% confidence intervals (95% CIs) were obtained as follows: (**Aa**) ranged from −0.254 to 0.31, (**Ab**) from −0.795 to −0.225, and (**Ac**) from 0.0316 to 0.710. (**Ba**) ranged from −0.555 to −0.0409, (**Bb**) from −0.799 to −0.236, and (**Bc**) from −0.519 to 0.273. (**Ca**) ranged from 0.00818 to 0.532, (**Cb**) from 0.109 to 0.746, and (**Cc**) from −0.548 to 0.235. (**Da**) ranged from 0.0779 to 0.580, (**Db**) from 0.0274 to 0.708, and (**Dc**) from −0.479 to 0.322. BCO1, β-carotene-15,15′-oxygenase; ER, estrogen receptor.

Correlation analyses were performed on *Nr1h4*, *Bco1*, and *Esr1* expression levels to elucidate the male-specific regulation of hepatic cholesterol metabolism by BC supplementation. Although no overall significant correlation was detected in all samples (n = 48, r = 0.0393, *p* = 0.824; Figure 4(Aa)), the mRNA expression levels of *Nr1h4* and *Bco1* exhibited a significant inverse correlation in males (n = 24, r = −0.576, *p* = 0.003; Figure 4(Ab)), however, they were positively correlated in the female groups (n = 24, r = 0.430, *p* = 0.036; Figure 4(Ac)). These findings indicated the correlation of *Nr1h4* and *Bco1* was sex-dependent. Moreover, a male-biased significant correlation was observed between mRNA expression levels of *Nr1h4* and *Esr1* (Figure 4(Bb)). After the potential relationship between hepatic BC cleavage enzymes and ERs was further investigated, *Bco1* had a significant positive correlation with the mRNA levels of *Esr1* and *Esr2* in all samples (n = 48, r = 0.292, *p* = 0.044; Figure 4(Ca), n = 48, r = 0.354, *p* = 0.014; Figure 4(Da)). When analyzed separately in terms of sex, this positive correlation remained significant only in males (n = 24, r = 0.490, *p* = 0.015; Figure 4(Cb), n = 24, r = 0.426, *p* = 0.038; Figure 4(Db)). Collectively, the male-specific effects of BC on ER expression, coupled with the observed correlation, indicated that hepatic cholesterol regulation upon BC supplementation might be mitigated by *Esr1* in males compared with that in females. These findings highlighted a possible link among cholesterol homeostasis, BC metabolism, and ER signaling. BCO1 was likely involved in orchestrating these male-specific associations.

### 2.5. Male-Specific Anti-Obesity Effects of BC on Gonadal Fat

**Figure 5:**
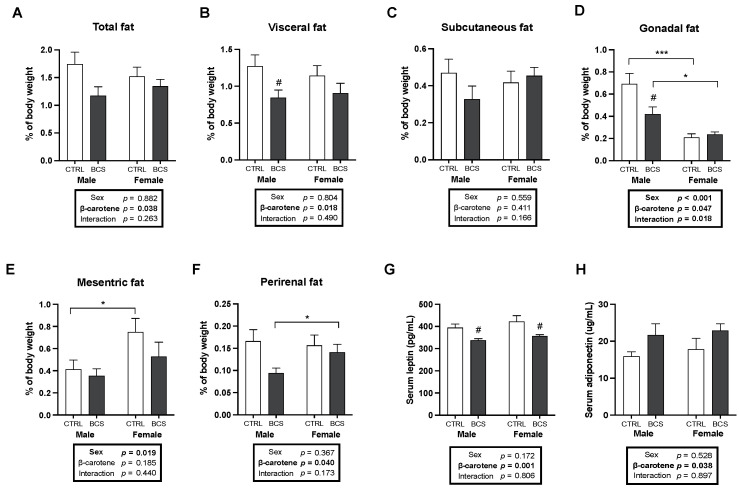
Male-specific anti-obesity effects of BC on gonadal fat. (**A**) Total fat mass (sum of mesenteric, perirenal, gonadal, and subcutaneous adipose tissues), (**B**) subcutaneous fat (n = 12/group), (**C**), visceral fat (sum of gonadal, mesenteric, and perirenal adipose tissues), (**D**) gonadal fat (n = 12/group), (**E**) mesenteric fat, and (**F**) perirenal fat mass (n = 12/group) in male and female mice (male CTRL: n = 12, male BCS: n = 11, female CTRL: n = 9, female BCS: n = 12 for total fat, visceral fat, and mesenteric fat). Serum levels of (**G**) leptin and (**H**) adiponectin were analyzed (n = 5/group). The normalization of all fat masses to body weight was calculated. All data were analyzed using two-way ANOVA with Tukey’s multiple comparisons. Results are presented as mean ± SEM. * for between sexes and # for between treatment groups (*, # *p* < 0.05, *** *p* < 0.001 in Student’s *t*-test or Welch’s *t*-test when applicable). BCS, group supplemented with 15 mg/kg body weight of β-carotene; CTRL, control group.

Fat distribution was compared to examine the sex-dependent effects of BC on adiposity. BC supplementation showed a tendency to decrease the total fat mass in males (Figure 5A). This trend was consistently found in various fat depots in males upon BC supplementation. Although subcutaneous fat mass displayed a non-significant decrease, visceral fat mass (the sum of gonadal, mesenteric, and perirenal adipose tissue weights) significantly decreased after BC supplementation in males (Figure 5B,C). Notably, gonadal fat exhibited significant male-specific effects of BC, with a significant interaction between sex and BC (Figure 5D). Because gonadal fat is in proximity to the gonads, it likely showed significant sex differences. To investigate whether the reduced adiposity affected circulating adipokines, such as serum leptin and adiponectin, levels were measured. In both sexes, BC supplementation significantly decreased leptin levels and non-significantly increased adiponectin levels (Figure 5G,H). These findings supported the effects of BC on reducing adiposity, particularly in males.

### 2.6. Male-Specific Decrease in BC Metabolism in Gonadal Adipose Tissue upon BC Supplementation

**Figure 6:**
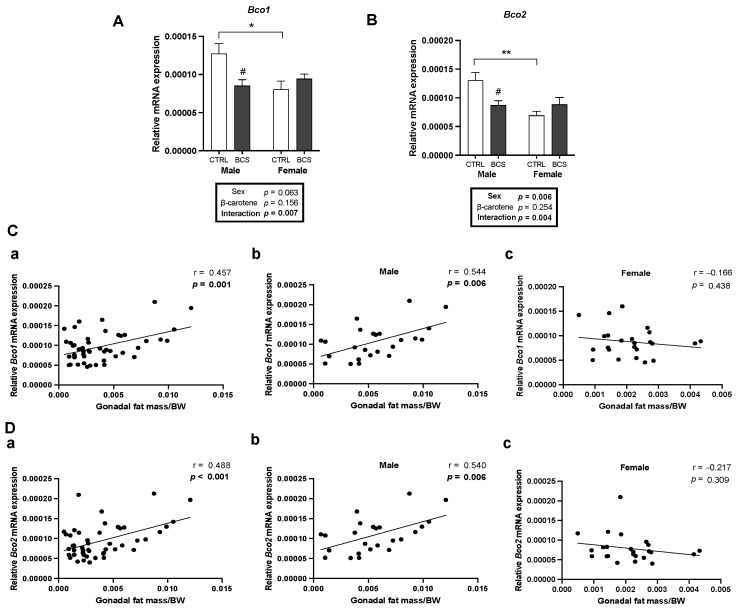
Male-specific decrease in BC metabolism in gonadal adipose tissue upon BC supplementation. mRNA expression of (**A**) *Bco1* and (**B**) *Bco2* in gonadal fat was analyzed by RT-qPCR analysis (n = 12/group). *Gapdh* was used as a loading control. All data were analyzed using two-way ANOVA with Tukey’s multiple comparisons. Results are presented as mean ± SEM. * for between sexes and # for between treatment groups (*, # *p* < 0.05, ** *p* < 0.01 in Student’s *t*-test). Correlation of gonadal fat mass/BW with mRNA expression of (**C**) *Bco1* and (**D**) *Bco2.* Pearson’s correlation coefficient was employed for statistical analysis. Correlations observed for the entire dataset as well as separately for male and female mice; (**a**) male + female, (**b**) male, and (**c**) female mice. Statistical significance was defined as *p* < 0.05. The 95% confidence intervals (95% CI) were obtained as follows: (**Ca**) ranged from 0.199 to 0.656, (**Cb**) from 0.180 to 0.777, and (**Cc**) from −0.534 to 0.254. (**Da**) ranged from 0.236 to 0.678, (**Db**) from 0.175 to 0.775, and (**Dc**) from −0.570 to 0.205. BCS, group supplemented with 15 mg/kg body weight of β-carotene; BW, body weight; CTRL, control group.

BC mainly accumulates in the adipose tissue, which expresses BCO1 and BCO2 [18]. In the present study, their mRNA levels were analyzed in gonadal fat to compare their expression patterns in adipose tissue and assess potential sex differences. Interestingly, in contrast to the hepatic expression patterns observed earlier, *Bco1* and *Bco2* mRNA expression levels were significantly higher in males than females (Figure 6A,B). BC supplementation significantly decreased *Bco1* and *Bco2* expression levels in male gonadal fat compared with those in the male CTRL group, with a significant interaction between sex and BC supplementation (Figure 6A,B). This finding was in contrast with the expression patterns in the liver. In the liver, BC significantly up-regulated the mRNA expression of *Bco1* and *Bco2* compared with that in the male CTRL group. However, the mRNA expression levels of BC cleavage enzyme genes were decreased by BC supplementation in the gonadal fat. These data suggested the contrasting *Bco1* and *Bco2* expression patterns between the liver and gonadal fat, indicating sex- and tissue-specific differences upon BC supplementation.

Pearson correlation analysis was conducted to determine which enzyme exerts a greater influence on gonadal fat reduction. The gonadal fat mass was positively correlated with the mRNA expression levels of *Bco1* and *Bco2* in all mice (n = 48, r = 0457, *p* = 0.001; Figure 6(Ca), n = 48, r = 0.488, *p <* 0.001, Figure 6(Da)). When analyzed separately by sex, this positive correlation remained significant only in males (n = 24, r = 0.544, *p* = 0.006; Figure 6(Cb), n = 24, r = 0.540, *p* = 0.006; Figure 6(Db)). These results demonstrated male-specific differences in gene levels at baseline and alterations by BC supplementation, indicating the distinct regulatory roles of BCO1 and BCO2 in the adipose tissue compared with those in the liver and a potential sex-dependent effect of BC on lipid metabolism in adipose tissue.

### 2.7. Male-Specific Decreases in Adipogenic and Lipogenic Markers in the Gonadal Adipose Tissue upon BC Supplementation

**Figure 7:**
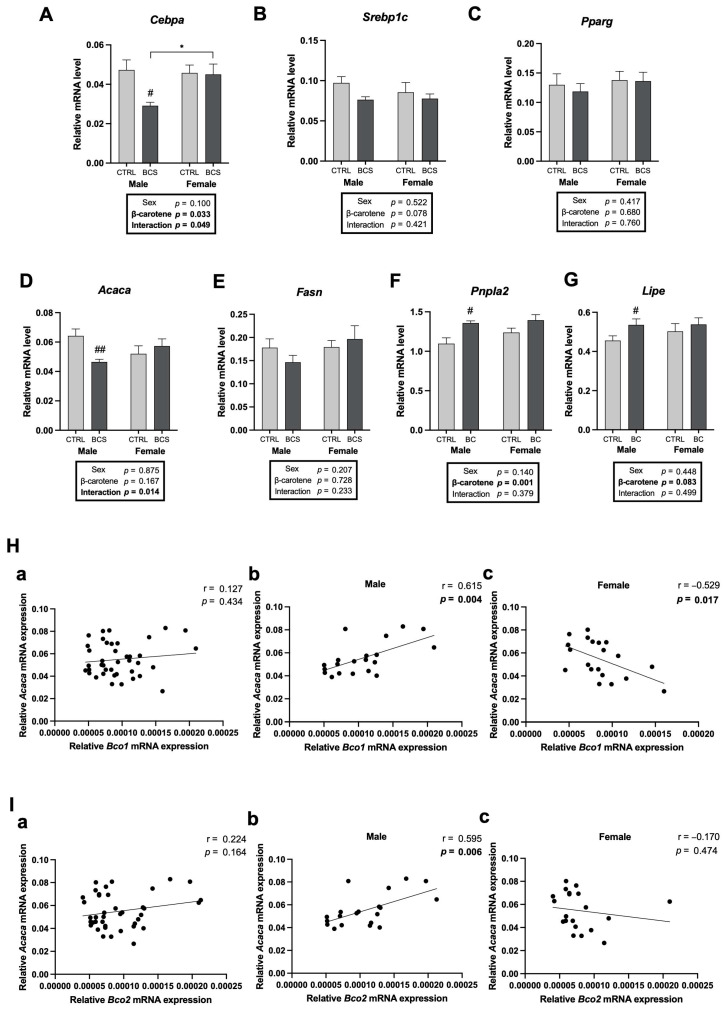
Male-specific decrease in adipogenic and lipogenic markers in the gonadal adipose tissue upon BC supplementation. mRNA expression of (**A**) *Cebpa*, (**B**) *Srebp1c*, (**C**) *Pparg*, (**D**) *Acaca*, (**E**) *Fasn*, (**F**) *Pnpla2*, and (**G**) *Lipe* were analyzed by RT-qPCR analysis (n = 10/group). All data were analyzed using two-way ANOVA with Tukey’s multiple comparisons. Results are presented as mean ± SEM. * for between sexes and # for between treatment groups (*, # *p* < 0.05, ## *p* < 0.01 in Student’s *t*-test). Correlation of (**H**) *Bco1* and (**I**) *Bco2* mRNA expression with *Acaca* (encoding ACC1). Pearson’s correlation coefficient was employed for statistical analysis. Correlations were observed for the entire dataset as well as separately for male and female mice; (**a**) male + female, (**b**) male, and (**c**) female mice. Statistical significance was defined as *p* < 0.05. The 95% confidence intervals (95% CI) were obtained as follows: (**Ha**) ranged from −0.192 to 0.422, (**Hb**) from 0.237 to 0.831, and (**Hc**) from −0.787 to −0.113. (**Ia**) ranged from −0.0936 to 0.501, (**Ib**) from 0.207 to 0.821, and (**Ic**) from −0.570 to 0.295. BCS, group supplemented with 15 mg/kg body weight of β-carotene; CTRL, control group.

The expression levels of adipogenic and lipogenic genes were analyzed to investigate the molecular mechanisms underlying the sex-dependent alterations in adiposity upon BC supplementation. C/EBPs, SREBP1c, and PPARγ are critical adipogenic markers. The mRNA expression levels of *Cebpa* significantly decreased in the male BCS group compared with that in the male CTRL group, exhibiting a significant interaction between sex and BC supplementation but no significant change in females (Figure 7A). The mRNA expression of *Srebp1c* decreased in the male BCS group compared with that in the CTRL group (Figure 7B). The expression of genes associated with fatty acid synthesis was assessed to support the decreasing trend of adipogenesis. BC supplementation significantly decreased the mRNA expression of *Acaca* (encoding ACC1) with a significant interaction between sex and BC supplementation, conversely, *Fasn* showed a non-significant decrease in males by BC supplementation (Figure 7D,E).

Lipolysis-related genes were examined further to understand the sex differences in lipid catabolism processes. BC supplementation significantly increased the mRNA expression levels of *pnpla2* (encoding Atgl) and *Lipe* (encoding HSL) in males, with significant BC main effects on *pnpla2* (Figure 7F,G). The potential relationship between BC metabolism and adipogenesis/lipogenesis was analyzed using the Pearson correlation. Although no significant correlation was found between the mRNA levels of *Acaca* and *Bco1* in all mice, including males and females (n = 40, r = 0.127, *p* = 0.434, Figure 7(Ha)), a significant and positive correlation was found when they were analyzed with the male group separately (n = 20, r = 0.615, *p* = 0.004; Figure 7(Hb)) and an inverse correlation with the female group (n = 20, r = −0.529, *p* = 0.017; Figure 7(Hc)). The mRNA expression levels of *Acaca* and *Bco2* in males had a significant positive correlation (n = 20, r = 0.595, *p* = 0.006; Figure 7(Ib)). The significant reduction in the mRNA expression of *Acaca* upon BC supplementation and the significant correlations with BC cleavage enzymes indicated that BC might regulate the lipogenic pathway in a sex-dependent manner.

### 2.8. Male-Specific Positive Correlation Between Expressions of ERs and Cebpa

**Figure 8:**
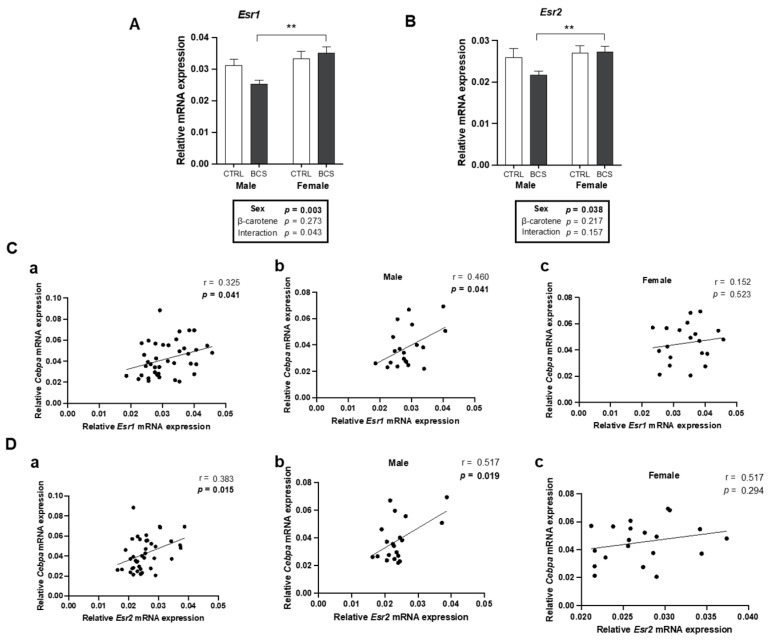
Male-specific ER expression and correlation with *Cebpa*. mRNA expressions of (**A**) *Esr1* (encoding ERɑ) and (**B**) *Esr2* (encoding ERβ) in the liver were analyzed by RT-qPCR analysis (n = 10/group). *Gapdh* was used as a loading control. All data were analyzed using two-way ANOVA with Tukey’s multiple comparisons. Results are presented as mean ± SEM. * for between sexes and # for between treatment groups (** *p* < 0.01 in Student’s *t*-test). Correlation of *Cebpa* mRNA expression with (**C**) *Esr1* and (**D**) *Esr2* mRNA expression. Pearson’s correlation coefficient was employed for statistical analysis. Correlations observed for the entire dataset as well as separately for male and female mice; (**a**) male + female, (**b**) male, and (**c**) female mice. Statistical significance was defined as *p* < 0.05. The 95% confidence intervals (95% CI) were obtained as follows: (**Ca**) ranged from 0.0145 to 0.578, (**Cb**) from 0.0223 to 0.750, and (**Cc**) from −0.312 to 0.557. (**Da**) ranged from 0.0811 to 0.620, (**Db**) from 0.0972 to 0.781, and (**Dc**) from −0.220 to 0.621. BCS, group supplemented with 15 mg/kg body weight of β-carotene: CTRL, control group.

The decrease in gonadal fat mass may be mediated by sex hormone signaling, considering its proximity to the gonads. Therefore, the mRNA expressions of *Esr1* (encoding ERɑ) and *Esr2* (encoding ERβ) were compared. The *Esr1* and *Esr2* expression levels were significantly higher in the female BCS group than in the male BCS group (Figure 8A,B). Interestingly, BC down-regulated the expression of ERs in males (Figure 8A,B). Pearson linear correlation was used to evaluate the potential roles of ERs in mediating BC-induced changes in lipid metabolism. *Cebpa* had a significant positive correlation with *Esr1* and *Esr2* in all mice (n = 40, r = 0.325, *p* = 0.041; Figure 8(Ca), n = 40, r = 0.383, *p* = 0.015; Figure 8(Da)). When analyzed separately by sex, this positive correlation remained significant solely in males (n = 20, r = 0.460, *p* = 0.041; Figure 8(Cb), n = 20, r = 0.517, *p* = 0.019; Figure 8(Db)). These results provided evidence supporting the potential role of ERs in mitigating the effects of BC on adipogenesis, specifically in males.

## 3. Discussion

BC supplementation elicited sex- and tissue-specific effects on lipid metabolism, in the liver and gonadal adipose tissue. Males exhibited a greater metabolic response to BC supplementation, characterized by significant up-regulation of hepatic BC metabolism and higher circulating retinol levels compared to females. Notably, BC supplementation reduced gonadal fat mass in males. These findings suggest that BC metabolism is tightly regulated by sex-specific mechanisms, potentially mediated by ER signaling and bile acid metabolism.

At baseline, females exhibited higher hepatic expression levels of *Bco1*, *Bco2*, *Rbp4*, and *Stra6l*, key markers of BC metabolism and transport. This suggests that females may have a more robust BC metabolism even in the absence of supplementation. In contrast, males demonstrated a more pronounced response to BC supplementation, with marked increases in hepatic expressions of BC cleavage enzymes (Bco1 and Bco2) and BC mobilization markers (Rbp4 and Stra6l), despite having lower baseline levels than females. Additionally, males exhibited higher circulating retinol levels upon BC supplementation compared to females, indicating a male-specific amplification of BC metabolism in response to supplementation.

A recent study observed that males have lower gut bioavailability of BC but higher systemic conversion efficiency to vitamin A than females [14]. This aligns with our findings, suggesting that males may compensate for their lower bioavailability by up-regulating hepatic BC metabolism and retinoid production. In contrast, females, with their higher baseline expression of BC metabolic markers, may have a reduced need for further BC metabolism. Furthermore, our preliminary analysis of public sequencing data revealed that the gene ontology term “fat-soluble vitamin metabolic process” was down-regulated in males compared to females in a BC-specific manner. Among the genes in this term, Lrp2 (encoding megalin), a key player in the endocytosis of retinol-binding protein, was expressed at lower levels in male liver compared to female liver (Supplementary Table S4) [19]. This down-regulation may result from negative feedback due to enhanced BC metabolite levels in the liver, potentially reducing hepatic capacity for further BC metabolism. This could also explain the observed higher circulating retinol levels in males, as circulating retinol primarily originates from the liver, which secretes it into the bloodstream in association with RBP4 [14].

Our findings suggest that BC may act as a central regulator of lipid metabolism through sex-specific mechanisms. A previous study highlighted BC metabolism is intricately linked to both tissue type and sex, with BCO1 playing a pivotal role [20]. While no significant differences in *Ar* mRNA expression were observed in the liver, sex-specific effects appear to be mediated by ER signaling, consistent with previous studies highlighting the critical role of estrogen in sex-specific lipid metabolism [7,10]. Although estrogens have traditionally been associated with female physiology, emerging evidence suggests that estrogen signaling may participate in regulating lipid metabolism in the liver of males [10,21]. The absence of *Esr1* leads to contrasting patterns between male and female mice in lipid deposition and metabolic profile [10]. In addition, in male gonadal fat, BC supplementation altered the expression of adipogenic genes, with a positive correlation observed between *Cebpa* and ER expression. Previous studies also demonstrated that estradiol can up-regulate adipogenesis through ERα-mediated CEBPα activation [7,22].

In this study, BC down-regulated the hepatic mRNA expression of *Nr1h4* in males, a key regulator of bile acid and lipid metabolism [23]. This down-regulation may reflect a compensatory response to reduced hepatic lipid levels, suggesting that BC modulates cholesterol metabolism and bile acid production in a sex-specific manner. Therefore, further studies should confirm their interactions and clarify the relationships among sex hormones, BC cleavage, and hepatic lipid metabolism.

Visceral fat is metabolically dynamic and highly influenced by adjacent tissues, as it encases critical organs [24]. Excess visceral fat is closely associated with metabolic diseases [9,25,26]. Among visceral fat depots, gonadal fat, a key depot of visceral fat, is the most readily accessible and widely studied, emphasizing its distinct metabolic role in visceral fat regulation. In the present study, gonadal fat exhibited the most pronounced sex differences and significant interactions with BC supplementation, indicating that it is the only fat depot where the combined effects of BC and sex were observed. This finding aligns with previous research reporting that male gonadal fat (epididymal fat) is the most obesity-responsive tissue in mice and plays a crucial role in metabolic disorders, including insulin sensitivity [27,28], particularly in response to dietary lipid-related stimuli. Notably, most studies investigating the relationship between gonadal fat and metabolic disease have primarily been conducted in male mice, which strongly aligns with our current findings. However, research in female mice suggests ERα is associated with low-grade inflammation and thermogenesis rather than adipogenesis in gonadal fat [29]. This may explain why the present study does not observe female-specific effects of BC compared to males, highlighting the need for further investigation in future studies.

Previous studies have also reported lipolysis is differentially regulated among different visceral fats under metabolic stress, such as high-fat diet exposure in male mice [30,31]. Notably, one study identified a compensatory response of visceral depot in fat mass [31], suggesting that different visceral fat depots may exhibit unique adaptations under metabolic challenges. Therefore, future studies are needed to elucidate the specific regulatory mechanisms underlying the sex-dependent effects of BC on lipid metabolism across various visceral fat depots.

Vitamin A is a critical factor for gonad development and spermatogenesis in males, with vitamin A deficiency leading to infertility [32,33,34]. In females, vitamin A deficiency can result in reproductive failure or fetal resorption, indicating its essential role in reproduction for both sexes. Furthermore, cellular retinol-binding protein (CRBP) levels are highest in the gonads compared to more than 20 tissues, including the liver, kidney, and epididymis [35]. It has also been reported that BC, as a local source of retinoids, plays a significant role in steroidogenic activity during the proliferation and differentiation of germinal cells in both males and females [36]. The storage and metabolism of BC and retinoids are tightly regulated in the ovary and granulosa cells, and in the testis, BC is converted into retinoic acid, which induces the proliferation of spermatogonia [37].

The present study defined visceral fat as the sum of gonadal, mesenteric, and perirenal adipose tissues. Based on the above information, among these depots, gonad fat was selected for analysis due to its sex differences, both within and between sexes, and its significant interaction effect with BC supplementation. Additionally, its proximity to the gonads allowed for the investigation of potential sex-specific regulatory effects. The significant reduction observed in healthy animals, without genetic manipulation or high-fat diet intervention, highlights its importance in understanding sex-specific differences in adipogenesis and lipid metabolism.

BC supplementation significantly reduced the male gonadal fat mass, indicating a male-specific anti-obesity effect. This aligns with the prior reports that sex hormones influence the spatial distribution of adipose tissue [38]. The observed reduction in gonadal fat may be the direct effect of BC, independent of its conversion to retinoids. Furthermore, the reduced expression levels of *Bco1* and *Bco2* in male gonadal fat imply that BC might directly regulate lipid metabolism in adipose tissue. This is consistent with the earlier study demonstrating that BC itself can inhibit lipid accumulation [39].

The dosage of 15 mg BC/kg body weight (b.w.) was selected based on our previous study, which showed the anti-colon cancer effect of BC at 15 mg/kg b.w. twice weekly [40] This dose corresponded to 4.2 mg/kg b.w./day in mice and 20.4 mg/day for a 60 kg human. In addition, 16 mg/kg b.w. BC inhibited tumor growth in prostate cancer xenograft mice [41]. It has also been reported that the plasma BC levels in nude mice administered with 16 mg BC/kg b.w. were 0.056 μM, which is comparable to the plasma level of 0.63 ± 0.52 μM observed in healthy humans [41].

This study has several limitations. The concentrations of BC and retinoids in various tissues were not comprehensively quantified due to sample size limitations, particularly for gonadal fat. Specifically, without diet interventions such as a high-fat diet, the low tissue weight of the gonadal adipose tissue samples (average 10 mg) presented a challenge; as such, this variable should be considered in future studies. Additionally, the effects of BC on other visceral fat depots, such as mesenteric and perirenal fat, were not examined. Exploring these depots could provide a more comprehensive understanding of the systemic effects of BC supplementation on visceral adipose tissue. However, several significant findings were obtained without genetic modifications in the animal model. Thus, the observed sex-specific effects of BC supplementation were robust and potentially translatable to other models or populations. Furthermore, this study provided insights into tissue-specific mechanisms by examining the effects of BC supplementation on multiple tissues, including the liver and gonadal adipose tissue.

## 4. Materials and Methods

### 4.1. Acquisition of Publicly Available Microarray Data

Microarray gene expression data were obtained from the Gene Expression Omnibus (GEO) database. After a 14-week dietary intervention with a control diet and a BC-supplemented diet (150 mg BC/kg diet), murine RNA samples from the liver were profiled using the Agilent-014868 (Agilent, Santa Clara, CA, USA) Whole Mouse Genome Microarray (G4122F) platform. GSE98846 was composed of 24 wild-type (WT) liver samples (12 male + 12 female), divided randomly into four groups (n = 6 per group) and fed with either a control diet or a BC-supplemented diet: (1) male mice with the control diet, (2) male mice with BC-supplemented diet, (3) female mice with the control diet, and (4) female mice with the BC-supplemented diet.

### 4.2. Screening for Differentially Expressed Genes (DEGs)

The raw data of GSE98846 were processed by log2 transformation and normalized using linear modeling. Specific coefficients for comparisons were extracted with the Limma package 3.62.2. The eBayes function was applied to implement the empirical Bayes method for stabilizing standard errors. DEGs between the male and female samples within the dataset were identified using the GEO2R online tool. Statistically significant genes were detected via a multiple *t*-test, followed by false discovery rate (FDR) correction. DEGs were selected under the following criteria: |log2 fold change| > 1 and FDR < 0.05. Gene ontology (GO) enrichment analysis was performed using the enrichGO function from the R package clusterProfiler 4.14.4.

### 4.3. Animal Experimental Design

Forty-eight 5-week-old BALB/c mice were purchased from Central Lab Animal Inc. (Seoul, Republic of Korea). After 1 week of acclimatization, the mice were randomly assigned to four experimental groups, housed in separate cages, with equal distribution based on both sex and diet to minimize potential environmental bias. The groups were as follows: male control (male CTRL, n = 12), male BC supplemented (male BCS, n = 12), female control (female CTRL, n = 12), and female BC supplemented (female BCS, n = 12). The experiment was conducted for 11 weeks under controlled environmental conditions (humidity: 50% ± 10%, temperature: 22 ± 1 °C, 12 h light/dark cycles). The mice were provided with water and chow ad libitum. The mice were fed the Teklad Global 18% Protein Rodent Diet (Envigo, Greenfield, IN, USA). Environmental parameters were monitored at least once a day to ensure adequate chow and water were provided for the mice.

The experimental groups were given 15 mg/kg b.w. BC dissolved in 100 μL of corn oil twice weekly via oral gavage, the control groups were administered with an equivalent volume of corn oil without BC supplementation. Prior to each gavage, the body weight of the mice was assessed. Based on the measured body weight, the BC concentration in the corn oil solution was recalculated and freshly prepared right before each administration to ensure the correct dosage.

The animal protocol for this study was approved by the Institutional Animal Care and Use Committee (IACUC) of Ewha Womans University (EWHA IACUC 22-063). Tissues were rinsed in phosphate-buffered saline, immediately frozen in liquid nitrogen during the sacrifice, and stored at −80 °C until analysis.

### 4.4. Assessment of Hepatic and Circulating Lipid Levels

Liver tissue samples were homogenized on ice, and lipids were extracted using the Bligh and Dyer chloroform–methanol solvent system protocol [42]. The chloroform (Sigma-Aldrich, St. Louis, MO, USA): methanol (JT Baker, Phillipsburg, NJ, USA) mixture (2:1, *v*/*v*) was vortexed and incubated for 30 min. After the mixture was vigorously shaken, additional chloroform was added and vortexed. After 2 min of incubation, distilled water was added, and the mixture was vortexed. Phase separation was performed via centrifugation (3000 rpm at 23 °C for 20 min). Then the lipid-containing organic phase was collected. This process was repeated twice. The lipid extract was poured into a filter paper funnel (Whatman qualitative grade 6, Whatman plc, Maidstone, UK), and the organic solvent was evaporated in a water bath at 80 °C for approximately 30 min. The filtered lipids were dried in an oven at 50 °C overnight and then cooled in a desiccator overnight and dissolved in chloroform:methanol (1:1, *v*/*v*). Triglyceride (TG) and cholesterol (TC) levels were measured using commercial assay kits (Asan Pharmaceutical Co., Ltd., Seoul, Republic of Korea).

### 4.5. Quantification of Circulating Retinol Concentration

Whole blood samples were collected, and serum was obtained through centrifugation (13,000 rpm at 4 °C for 15 min) and stored at −80 °C until analysis. Retinol and retinyl acetate were used as external and internal standards, respectively. Serum samples for high-performance liquid chromatography (HPLC) analysis were prepared by combining 10 µL of serum samples and 10 µL of internal standard (1 µM retinyl acid). Then, 10 µL of ethanol (Avantor, Randor, PA, USA) was added to precipitate the proteins in the serum, and 100 µL of n-hexane (Avantor) was added to extract non-polar compounds from the serum and vortexed vigorously for 10 min. The extraction mixtures were centrifuged at 1500× *g* and 25 °C for 5 min. The supernatant hexane layer was transferred to a new tube and dried under nitrogen flow at 40 °C in a heating block. The dry pellet was reconstituted in 10 μL of the mobile phase. An injection volume of 2 μL was used for each analysis.

HPLC analysis was conducted using a Nanospace instrument (Shiseido, Tokyo, Japan) equipped with an SP3002 UV detector, an SP3101 pump, an SP3023 injector, and an SI-2 3004 column oven. The mobile phase comprised 0.1% phosphoric acid, methanol, and acetonitrile (15:42.5:42.5, *v*/*v*/*v*; JT Baker). All devices mentioned were calibrated prior to analysis by a certified technician from the HPLC machine supplier, ensuring their proper functionality and accuracy of parameters such as flow rate and oven temperature. It was pumped into a C18 MG2 column (Osaka Soda Co., Osaka, Japan) at a flow rate of 200 μL/min. The oven temperature was maintained at 40 °C to ensure proper elution and separation. Retinol was detected at 325 nm, and concentrations were quantified based on the peak area ratios of retinol and retinyl acetate. For each run, standard curves were prepared using retinol concentrations ranging from 0.015 ppm to 0.5 ppm, with linearity confirmed by an R^2^ value consistently exceeding 0.98, indicating strong correlation within this range. All measured values fell within the standard curve, ensuring accurate and reliable quantification.

### 4.6. RNA Isolation and Gene Expression Analysis

Total RNA was extracted from tissue samples using TRIzol™ Reagent (Thermo Fisher Scientific, Waltham, MA, USA) according to the manufacturer’s instructions. The isolated RNA was reverse transcribed to complementary DNA (cDNA) using the RevertAid First Strand cDNA Synthesis kit (Thermo Fisher Scientific). Quantitative real-time PCR was performed using the SYBR Green PCR master mix (Bioneer, Daejeon, Republic of Korea) on a Rotor-Gene Q real-time PCR cycler (Qiagen, Hilden, Germany). The expression levels of target genes were normalized to the housekeeping gene *Gapdh*.

### 4.7. Western Blots

Liver tissue samples were lysed in a PRO-PREP Protein extraction solution (iNtRON Biotechnology, Seongnam, Republic of Korea) supplemented with a phosphatase inhibitor cocktail (Sigma-Aldrich). Protein concentrations were determined using a Bio-Rad protein assay kit (Bio-Rad, Hercules, CA, USA). Equal amounts of proteins were resolved via sodium dodecyl sulfate–polyacrylamide gel electrophoresis (SDS-PAGE) and transferred to polyvinylidene fluoride (PVDF) membranes (Milipore, Billerica, MA, USA). The membranes were blocked with 5% skim milk (GeorgiaChem, Norcross, GA, USA) in Tris-buffered saline (TBS; Tris-Cl, NaCl, pH 7.5) with 0.05% Tween 20 (Bio-Rad) and incubated with primary antibodies diluted in the blocking solution at 4 °C overnight. Primary antibodies were anti-BCO1 (ABclonal, Woburn, MA, USA), anti-BCO2 (Proteintech, Rosemont, IL, USA), anti-ERɑ (Santa Cruz Biotechnology, Inc., Dallas, TX, USA), anti-ERβ (GeneTex, Irvine, CA, USA), GAPDH (Invitrogen, Waltham, CA, USA), and β-actin (Abcam, Cambridge, UK). After washing, the membranes were incubated with appropriate anti-mouse (Rockland Immunochemicals, Rockland, NY, USA) or anti-rabbit (Santa Cruz Biotechnology) secondary antibodies. Protein bands were visualized using an enhanced chemiluminescence detection system (Animal Genetics Inc., Suwon, Republic of Korea). Band intensities were quantified using ImageJ software 1.53t (US National Institutes of Health, MD, USA).

### 4.8. Enzyme-Linked Immunosorbent Assay (ELISA) 

The levels of leptin (mouse leptin ELISA kit; LEP) and adiponectin (mouse adiponectin ELISA kit; ADP) in the mouse serum samples were measured using ELISA kits (ABclonal, Woburn, MA, USA) following the manufacturer’s instructions.

### 4.9. Statistical Analysis

Data were presented as mean ± standard error of the mean (SEM) and analyzed using GraphPad PRISM 9 (GraphPad Software, San Diego, CA, USA). Relationships between markers among different groups were investigated via Pearson correlation analysis. Data with *p* < 0.05 were considered statistically significant. Significant differences between the two groups were also analyzed using Student’s *t*-test (Microsoft 365, Redmond, WA, USA). Welch’s *t*-test was used when the assumption of equal variance was not met. The effects of sex and BC supplementation were assessed via two-way analysis of variance (ANOVA) with Tukey’s multiple comparisons test.

## 5. Conclusions

This study described the intricate relationship between BC metabolism, ER signaling, and lipid regulation in a sex-dependent manner. We observed that overall adiposity was consistently decreased in male mice. BC increased the expression of BC cleavage enzymes in the liver of male mice but decreased their expression in gonadal fat. The reduction in gonadal fat mass in males might have been mediated by the direct effects of BC regardless of its conversion to retinoids. Taken together, this study demonstrated the sex- and tissue-specific effects of BC supplementation on lipid metabolism in the liver and gonadal fat. The findings suggest that BC acts as a central regulator of lipid metabolism, exerting male-specific effects through ER signaling and modulating bile acid metabolism. The observed sex-specific responses, identified in a non-genetically modified model, may be translatable to other models or populations. These results provide valuable insights into the interplay between BC metabolism, sex hormones, and lipid/cholesterol metabolism, emphasizing the significance of tissue- and sex-specific approaches in nutritional research.

## Data Availability

The datasets are available from the corresponding author upon reasonable request with permission.

## Supplementary Materials

The following supporting information can be downloaded at: https://www.mdpi.com/article/10.3390/molecules30040909/s1; Figure S1: GO analysis of DEGs in the liver upon BC supplementation. Table S1: The primer sequence used in RT-qPCR. Table S2: Composition of the chow diet. Table S3: Results of Gene Ontology (GO) enrichment analysis of upregulated genes identified in the male vs. female under the BC-supplemented diet. Table S4: Results of Gene Ontology (GO) enrichment analysis of downregulated genes identified in the male vs. female under the BC-supplemented diet.
