# Supplementary material for: Male-Specific Effects of β-Carotene Supplementation on Lipid Metabolism in the Liver and Gonadal Adipose Tissue of Healthy Mice
Source: Molecules. 2025 Feb 15;30(4):909. doi: 10.3390/molecules30040909 (PMC11858425; doi:10.3390/molecules30040909)
Supplement: Supplementary file 1 [file molecules-30-00909-s001.zip › Supplementary Figure Legend (revision final).pdf]

Supplementary Figure Legend

Supplementary Figure S1. GO analysis of DEGs in the liver upon BC supplementation.

B6129SF1 mice (n=6/group) were fed control or BC diets containing water-soluble beadlets (150 mg BC/kg diet) for 14 weeks starting at 5 weeks of age. (A) Venn diagrams of DEGs identified in the liver from the comparisons: 1) Control diet-fed males vs. females (Liver WT CTRL M vs CTRL F) and 2) BC-treated males vs. BC-treated females (Liver WT BC M vs WT BC F). The 86 DEGs were analyzed as the sex-specific DEGs on BC supplementation in the liver. (B) The top 5 GO terms for genes differentially expressed by BC supplementation in a sex-specific manner in the liver. a) Top 5 GO pathway terms enriched in a) up-regulated DEGs and b) down-regulated DEGs by BC supplementation in male livers.

BC, β-carotene; CTRL, control; DEGs, differentially expressed genes; F, female; M, male; GO, Gene Ontology; WT, wildtype
