# Supplementary material for: Male-Specific Effects of β-Carotene Supplementation on Lipid Metabolism in the Liver and Gonadal Adipose Tissue of Healthy Mice
Source: Molecules. 2025 Feb 15;30(4):909. doi: 10.3390/molecules30040909 (PMC11858425; doi:10.3390/molecules30040909)
Supplement: Supplementary file 1 [file molecules-30-00909-s001.zip › Supplementary Table S1 (revised final).pdf]

Supplementary Table S1. The primer sequence used in RT-qPCR.

Gene name	Gene ID	Primer sequence (5’→3’)
Bco1	63857	F: GAGCAGCCTTTTAAGTTGGA<br>R: GGTCCTCTGGTCGATGATAT
Bco2	170752	F: TACAACATGGGGAACAGCTA<br>R: GGCTTCATTTTCTCAGTGGA
Rbp4	19662	F: TGTAGCCTCCTTTCTCCAGCGA<br>R: ACAGGTGCCATCCAGATTCTGC
Stra6l	74152	F: GCTGGCAGATAGCCTACATCCT<br>R: CCAAATCCTTGCAGCATCTCCAG
Esr1	13982	F: TCTGCCAAGGAGACTCGCTCCT<br>R: GGTGCATTGGTTTGTAGCTGGAC
Esr2	13983	F: GGTCCTGTGAAGGATGTAAGGC<br>R: TAACACTTGCGAAGTCGGCAG
Ar	11835	F: CCTTGGATGGAGAACTACTCCG<br>R: TCCGTAGTGACAGCCAGAAGCT
Ldlr	16835	F: GAATCTACTGGTCCGACCTGTC<br>R: CTGTCCAGTAGATGTTGCGGTG
Nr1h4	20186	F: GGGATGAGTGTGAAGCCAGCTA<br>R: GTGGCTGAACTTGAGGAAACGG
Cebpa	12606	F: CCAAGAAGTCGGTGGACAAGA<br>R: CGGTCATTGTCACTGGTCAACT
Pparg	19016	F: AAGAGCTGACCCAATGGTTG<br>R: TGAGGCCTGTTGTAGAGCTG
Acaca	107476	F: CACAGTGCTCAAAGGACATGCC<br>R: CACCAGGTGTAGTGCCTTCCTC
Fasn	14104	F: GTTCTGTTGGACAACGCCTTCAC<br>R: GGAGTCACAGAAGCAGCCCATT
Pnpla2	66853	F: GGAACCAAAGGACCTGATGACC<br>R: ACATCAGGCAGCCACTCCAACA
Lipe	16890	F: GCTCATCTCCTATGACCTACGG<br>R: TCCGTGGATGTGAACAACCAGG

Ppargc1a	19017	F: GAATCAAGCCACTACAGACACCG<br>R: CATCCCTCTTGAGCCTTTCGTG
Ucp1	22227	F: GCTTTGCCTCACTCAGGATTGG<br>R: CCAATGAACACTGCCACACCTC
Gapdh	14433	F: AACTTTGGCATTGTGGAAGG<br>R: TGTGAGGGAGATGCTCAGTG
