# Supplementary material for: Male-Specific Effects of β-Carotene Supplementation on Lipid Metabolism in the Liver and Gonadal Adipose Tissue of Healthy Mice
Source: Molecules. 2025 Feb 15;30(4):909. doi: 10.3390/molecules30040909 (PMC11858425; doi:10.3390/molecules30040909)
Supplement: Supplementary file 1 [file molecules-30-00909-s001.zip › Supplementary Table S2 (revision final).pdf]

Supplementary Table S2. Composition of the chow diet.

**Macronutrients**	
Crude Protein	18.6%
Fat (ether extract)	6.2%
Carbohydrate	44.2%
Crude Fiber	3.5%
Neutral Detergent Fiber	14.7%
Ash	5.3%
Energy Density	3.1 kcal (13.0 kJ/g)
Calories from Protein	24%
Calories from Fat	18%
Calories from Carbohydrate	58%
**Minerals**	
Calcium	1.0%
Phosphorus	0.7%
Non-Phytate Phosphorus	0.4%
Sodium	0.2%
Potassium	0.6%
Chloride	0.4%
Magnesium	0.2%
Zinc	70 mg/kg
Manganese	100 mg/kg
Copper	15 mg/kg
Iodine	6 mg/kg
Iron	200 mg/kg
Selenium	0.23 mg/kg

Amino acids	
Aspartic Acid	1.4%
Glutamic Acid	3.4%
Alanine	1.1%
Glycine	0.8%
Threonine	0.7%
Proline	1.6%
Serine	1.1%
Leucine	1.8%
Isoleucine	0.8%
Valine	0.9%
Phenylalanine	1.0%
Tyrosine	0.6%
Methionine	0.4%
Cystine	0.3%
Lysine	0.9%
Histidine	0.4%
Arginine	1.0%
Tryptophan	0.2%

Vitamins	
Vitamin A*	15.0 IU/g
Vitamin D3	1.5 IU/g
Vitamin E	110 IU/kg
Vitamin K3 (menadione)	50 mg/kg
Vitamin B1 (thiamin)	17 mg/kg
Vitamin B2 (riboflavin)	15 mg/kg
Niacin (nicotinic acid)	70 mg/kg

**Vitamins**	
Vitamin B6 (pyridoxine)	18 mg/kg
Pantothenic Acid	33 mg/kg
Vitamin B12 (cyanocobalamin)	0.08 mg/kg
Biotin	0.40 mg/kg
Folate	4 mg/kg
Choline	1200 mg/kg
**Fatty acids**	
C16:0 Palmitic	0.7
C18:0 Stearic	0.2
C18:1ω9 Oleic	1.2
C18:2ω6 Linoleic	3.1
C18:3ω3 Linolenic	0.3
Total Saturated	0.9
Total Monounsaturated	1.3
Total Polyunsaturated	3.4
**Others**	
Cholesterol	-

* 1 IU vitamin A = 0.3 µg retinol
