# Supplementary material for: Male-Specific Effects of β-Carotene Supplementation on Lipid Metabolism in the Liver and Gonadal Adipose Tissue of Healthy Mice
Source: Molecules. 2025 Feb 15;30(4):909. doi: 10.3390/molecules30040909 (PMC11858425; doi:10.3390/molecules30040909)
Supplement: Supplementary file 1 [file molecules-30-00909-s001.zip › Supplementary Table S3 (revision final).pdf]

Supplementary Table S3. Results of Gene Ontology (GO) enrichment analysis of upregulated genes identified in the male vs. female under the BC-supplemented diet.

ID	Description	GeneRatio	Fold Enrichment	*P* value	Gene name	Count
GO:0060749	mammary gland alveolus development	3/35	112.62	2.E-06	Esr 1/Socs2/Id 2	3
GO:0061377	mammary gland lobule development	3/35	112.62	2.E-06	Esr 1/Socs2/Id 2	3
GO:0006706	steroid catabolic process	3/35	103.24	3.E-06	Cyp4a31	3
GO:0045833	negative regulation of lipid metabolic process	4/35	27.76	1.E-05	Esr 1/Fmo 1/Fmo4/Cyp4a31	4
GO:0045922	negative regulation of fatty acid metabolic process	3/35	63.53	1.E-05	Fmo 1/Fmo4/Cyp4a31	3
GO:0042832	defense response to protozoan	3/35	57.62	2.E-05	Gbp7/Irgm2/gbp11	3
GO:0001562	response to protozoan	3/35	51.62	3.E-05	Gbp7/Irgm2/gbp11	3
GO:1901616	organic hydroxy compound catabolic process	3/35	38.71	6.E-05	Cyp4a31	3
GO:0019216	regulation of lipid metabolic process	5/35	10.59	1.E-04	Esr1/Fmo 1/Fmo4/Cyp4a31	5
GO:0030258	lipid modification	4/35	15.58	1.E-04	Fmo 1/Plppr1/Fmo4/Hacl1	4
GO:0061762	CAMKK-AMPK signaling cascade	2/35	117.99	1.E-04	Camk2	2
GO:0046322	negative regulation of fatty acid oxidation	2/35	91.77	2.E-04	Fmo 1/Fmo4	2
GO:0006631	fatty acid metabolic process	5/35	8.64	3.E-04	Fmo 1/Fmo4/Cyp4a31	5
GO:0042180	cellular ketone metabolic process	4/35	12.47	3.E-04	Fmo 1/Fmo4/Cyp4a31	4
GO:0019217	regulation of fatty acid metabolic process	3/35	21.93	3.E-04	Fmo 1/Fmo4/Cyp4a31	3
GO:0071346	cellular response to type II interferon	3/35	21.00	4.E-04	Gbp7/Irgm2/gbp11	3

ID	Description	GeneRatio	Fold Enrichment	*P* value	Gene name	Count
GO:0019395	fatty acid oxidation	3/35	20.48	4.E-04	Fmo 1/Fmo4/Hacl1	3
GO:0034440	lipid oxidation	3/35	19.21	5.E-04	Fmo 1/Fmo4/Hacl1	3
GO:0062014	negative regulation of small molecule metabolic process	3/35	19.06	5.E-04	Fmo 1/Fmo4/Cyp4a31	3
GO:0046426	negative regulation of receptor signaling pathway via JAK-STAT	2/35	58.99	5.E-04	Gbp7/Socs2	2
GO:0045648	positive regulation of erythrocyte differentiation	2/35	56.96	6.E-04	Isg15/Id2	2
GO:1904893	negative regulation of receptor signaling pathway via STAT	2/35	53.28	6.E-04	Gbp7/Socs2	2
GO:0032479	regulation of type I interferon production	3/35	17.57	7.E-04	Gbp7/Isg15/Irgm2	3
GO:0016053	organic acid biosynthetic process	4/35	9.95	7.E-04	Fmo 1/Cyp4a31	4
GO:0006699	bile acid biosynthetic process	2/35	51.62	7.E-04	Cyp4a31	2
GO:0034341	response to type II interferon	3/35	17.33	7.E-04	Gbp7/Irgm2/gbp11	3
GO:0032606	type I interferon production	3/35	16.97	7.E-04	Gbp7/Isg15/Irgm2	3
GO:0033598	mammary gland epithelial cell proliferation	2/35	50.06	7.E-04	Esr1/Id2	2
GO:0016042	lipid catabolic process	4/35	9.49	8.E-04	Cyp4a31	4
GO:0008202	steroid metabolic process	4/35	9.03	1.E-03	Esr1/Cyp4a31	4
GO:0001822	kidney development	4/35	8.81	1.E-03	Bmper/Prom1/Id2/Cyp4a31	4
GO:0044282	small molecule catabolic process	4/35	8.79	1.E-03	Cyp4a31	4
GO:0010565	regulation of cellular ketone metabolic process	3/35	14.41	1.E-03	Fmo 1/Fmo4/Cyp4a31	3
GO:0046164	alcohol catabolic process	2/35	39.33	1.E-03	Cyp4a31	2

ID	Description	GeneRatio	Fold Enrichment	*P* value	Gene name	Count
GO:0072001	renal system development	4/35	8.47	1.E-03	Bmper/Prom1/Id2/Cyp4a31	4
GO:0030879	mammary gland development	3/35	14.00	1.E-03	Esr1/Socs2/Id2	3
GO:0032480	negative regulation of type I interferon production	2/35	36.71	1.E-03	Gbp7/Irgm2	2
GO:0046320	regulation of fatty acid oxidation	2/35	36.71	1.E-03	Fmo 1/Fmo4	2
GO:0042742	defense response to bacterium	4/35	8.00	2.E-03	Gbp7/Isg15/Irgm2/gbp11	4
GO:0015718	monocarboxylic acid transport	3/35	12.77	2.E-03	Fabp12/Cyp4a31	3
GO:0008206	bile acid metabolic process	2/35	33.04	2.E-03	Cyp4a31	2
GO:0045646	regulation of erythrocyte differentiation	2/35	32.39	2.E-03	Isg15/Id2	2
GO:0043124	negative regulation of canonical NF-kappaB signal transduction	2/35	26.64	3.E-03	Esr1/Irgm2	2
