# Supplementary material for: Male-Specific Effects of β-Carotene Supplementation on Lipid Metabolism in the Liver and Gonadal Adipose Tissue of Healthy Mice
Source: Molecules. 2025 Feb 15;30(4):909. doi: 10.3390/molecules30040909 (PMC11858425; doi:10.3390/molecules30040909)
Supplement: Supplementary file 1 [file molecules-30-00909-s001.zip › Supplementary Table S4 (revision final).pdf]

Supplementary Table S4. Results of Gene Ontology (GO) enrichment analysis of downregulated genes identified in the male vs. female under the BC-supple-mented diet.

ID	Description	GeneRatio	Fold Enrichment	*P* value	Gene name	Count
GO:0010544	negative regulation of platelet activation	2/25	144.54	8.6E-05	Serpin	2
GO:0014013	regulation of gliogenesis	3/25	24.09	2.6E-04	Serpin	3
GO:0048608	reproductive structure development	4/25	11.98	3.2E-04	Serpin	4
GO:0061458	reproductive system development	4/25	11.83	3.3E-04	Serpin	4
GO:0046660	female sex differentiation	3/25	20.53	4.1E-04	Lrp2/Afp/Fst	3
GO:0002053	positive regulation of mesenchymal cell proliferation	2/25	66.07	4.2E-04	Pdgf	2
GO:0006775	fat-soluble vitamin metabolic process	2/25	60.86	5.0E-04	Lrp2/Cyp26a1	2
GO:0048710	regulation of astrocyte differentiation	2/25	60.86	5.0E-04	Serpin	2
GO:0010544	negative regulation of platelet activation	2/25	144.54	8.6E-05	Serpin	2
GO:0014013	regulation of gliogenesis	3/25	24.09	2.6E-04	Serpin	3
GO:0048608	reproductive structure development	4/25	11.98	3.2E-04	Serpin	4
GO:0061458	reproductive system development	4/25	11.83	3.3E-04	Serpin	4
