# Supplementary figures and images for: Male-Specific Effects of β-Carotene Supplementation on Lipid Metabolism in the Liver and Gonadal Adipose Tissue of Healthy Mice
Source: Molecules. 2025 Feb 15;30(4):909. doi: 10.3390/molecules30040909 (PMC11858425; doi:10.3390/molecules30040909)

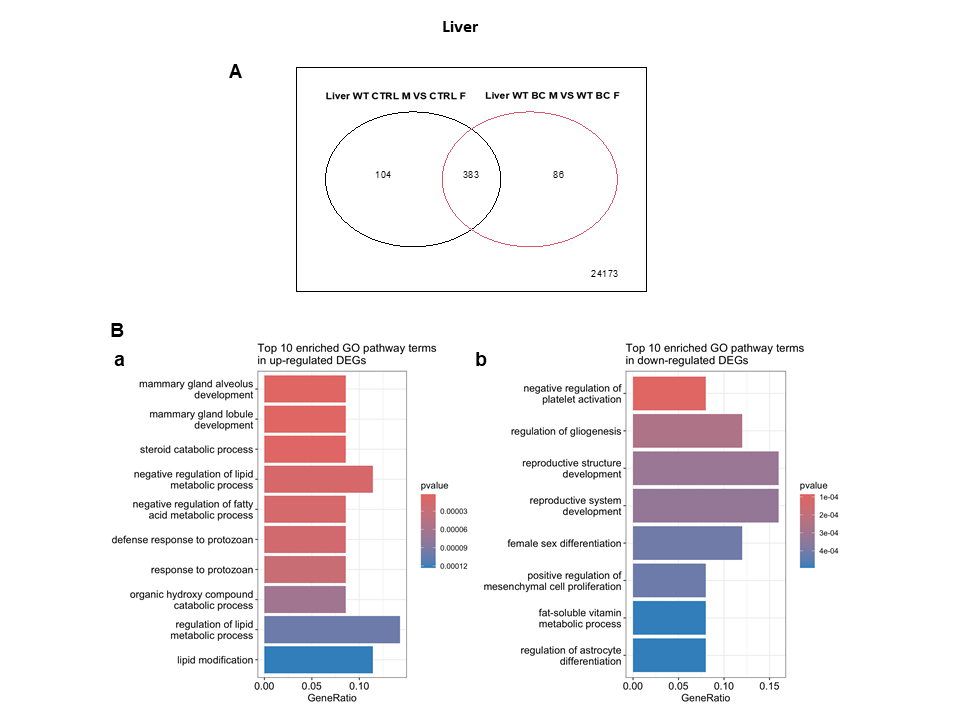

Supplement: Supplementary file 1 [file molecules-30-00909-s001.zip › Supplementary Figure S1.tif]
